# Computational modeling of heterogeneity and function of CD4+ T cells

**DOI:** 10.3389/fcell.2014.00031

**Published:** 2014-07-29

**Authors:** Adria Carbo, Raquel Hontecillas, Tricity Andrew, Kristin Eden, Yongguo Mei, Stefan Hoops, Josep Bassaganya-Riera

**Affiliations:** ^1^Nutritional Immunology and Molecular Medicine Laboratory, Virginia Bioinformatics Institute, Virginia TechBlacksburg, VA, USA; ^2^Center for Modeling Immunity to Enteric Pathogens, Virginia Bioinformatics Institute, Virginia TechBlacksburg, VA, USA; ^3^Department of Biomedical Sciences and Pathobiology, Virginia-Maryland Regional College of Veterinary Medicine, Virginia TechBlacksburg, VA, USA

**Keywords:** CD4+ T cell differentiation, computational modeling, immunoinformatics, computational immunology, CD4+ T cell dogma

## Abstract

The immune system is composed of many different cell types and hundreds of intersecting molecular pathways and signals. This large biological complexity requires coordination between distinct pro-inflammatory and regulatory cell subsets to respond to infection while maintaining tissue homeostasis. CD4+ T cells play a central role in orchestrating immune responses and in maintaining a balance between pro- and anti- inflammatory responses. This tight balance between regulatory and effector reactions depends on the ability of CD4+ T cells to modulate distinct pathways within large molecular networks, since dysregulated CD4+ T cell responses may result in chronic inflammatory and autoimmune diseases. The CD4+ T cell differentiation process comprises an intricate interplay between cytokines, their receptors, adaptor molecules, signaling cascades and transcription factors that help delineate cell fate and function. Computational modeling can help to describe, simulate, analyze, and predict some of the behaviors in this complicated differentiation network. This review provides a comprehensive overview of existing computational immunology methods as well as novel strategies used to model immune responses with a particular focus on CD4+ T cell differentiation.

## Introduction

The human immune system consists of two main behavioral and functional waves: first, the innate immune response provides a first barrier against foreign elements and second, the adaptive immune system builds an effective and specific immune response to combat such elements. The principal function of the adaptive responses is not only the specific recognition to foreign antigens, but also the formation of immunologic memory, and the development of tolerance to self-antigens (Luckheeram et al., 2012). Originated in the bone marrow and matured in the thymus, CD4+ T cells are part of the specific adaptive immunity compartment. T cell selection in the thymus allows creating an array of T cell repertoire for antigen recognition, as well as allowing the selection process through MHC-II and the expression of surface markers, such as CD4 or CD8 (Klein et al., 2009). Mature CD4+ T cells then translocate into the secondary lymphoid organs, such as the lymph nodes or the spleen, where they are involved in immune surveillance through interaction with MHC-II molecules expressed on the surface of antigen-presenting cells (Drayton et al., 2006). In this inductive site, naïve CD4+ T cells sample the tissue environment and depending on the cytokine milieu, they differentiate into functionally distinct regulatory or effector subsets.

The central dogma of CD4+ T cell differentiation has evolved over the past decades as new studies have unveiled differentiation pathways and novel mechanisms shaping the CD4+ T cell compartment. The Th1 vs. Th2 conceptual framework that Mossman and Coffman provided (Mosmann and Coffman, 1989) was largely expanded when novel discoveries on RORγt and IL-17A producing T cells defined the Th17 phenotype (Ivanov et al., 2006) and with the identification of FOXP3 raised as a key transcription factor in charge of driving the regulatory response in CD4+ T cells (Fontenot et al., 2003; Hori et al., 2003). Recent in-depth characterization of CD4+ T cell lineages has resulted in the discovery of new phenotypes, positioning the CD4+ T cell population as one of the most heterogeneous immune cell subsets. Furthermore, the latest discoveries are pushing the understanding of CD4+ T cell differentiation from a 4-player game to a multi-pronged interplay of complex networks and common transcription factors and cytokines with highly plastic functionalities. As an example, the production of IL-9 by the transcription factor PU.1 leads to the establishment of the Th9 phenotype (Ma et al., 2010). Furthermore, other phenotypes, such as Th17, are now under scrutiny since IL-17 and IL-22 are co-expressed in an IL-23 dependent manner (Trifari and Spits, 2010; Sonnenberg et al., 2011). New studies are pointing out to the aryl hydrocarbon receptor (AhR) as the master transcription factor responsible for IL-22 secretion (Ramirez et al., 2010), leading to the designation of a new CD4+ T cell phenotype, Th22, which has been also identified in humans (Eyerich et al., 2009; Fujita et al., 2009) Moreover, FOXP3-independent IL-10 upregulation has been implicated in the activation of the regulatory axis under the regulatory type 1 (Tr1) CD4+ T cells (Pot et al., 2011). Lastly, follicular T helper cells (Tfh) have become an object of intense study since they have been described as a very plastic subset that could swift the CD4+ T cell balance. Tfh cells can leave the T cell areas and localize in the B cell follicle, a migration that is facilitated by their concurrent expression of the B cell zone homing chemokine receptor CXCR5 and downregulation of the T cell zone homing chemokine receptor CCR7 (Ansel et al., 1999; Hardtke et al., 2005). Thus, this close proximity to B cells allows Tfh cells to support their activation, expansion and differentiation. To help promote this crosstalk with B cells, Tfh cells produce IL-21 via activation of the transcription factor BCL-6, thereby promoting a Th1/Th17 profile. Also, IL-2 is emerging as a trigger for Th1 differentiated cells to adopt a Tfh-like phenotype by down-regulating BLIMP1 and interacting with STAT proteins (Breitfeld et al., 2000). Since the BCL-6 pathway is linked to STAT factors induced by IL-6 that in turn promotes IL-21 and TNFα production, the study of the role of Tfh is important in the context of infectious, immune-mediated, or chronic inflammatory diseases.

Computational modeling has become an indispensable tool to synthesize, organize, and integrate diverse data types and theoretical frameworks to help generate new knowledge and guide *in vivo* experimentation. This review highlights how computational modeling has helped advancing the understanding of signaling events controlling CD4+ T heterogeneity and it also discusses new opportunities in the context of modeling strategies and tools.

## Mathematical modeling and CD4+ T cell differentiation

Initial attempts to apply computational modeling approaches to study CD4+ T cell differentiation only focused on the Th1 and Th2 phenotypes. Indeed the well-established dichotomy between these two phenotypes is supported by extensive information on how T-bet (Th1) and GATA3 (Th2) interact. One of the first published studies extrapolated the Th1/Th2 experimental facts into systemic behavior during an immune response, indicating that suppression and domination of one phenotype over the other could dictate the final differentiation outcome (Fishman and Perelson, 1999). In this study, the model encompassed not only Th1 and Th2, but also the effect of antigen presentation via APCs. This mathematical model illustrated how the final differentiation of Th1 or Th2 depends in both the competition for antigenic stimulation and the cytokine-mediated cross suppression between phenotypes. Subsequent studies applied mathematical modeling to study the Th1 and Th2 phenotypes in the presence of other cytokines such as IL-10 or TGFβ (Yates et al., 2000), antigen availability and instructional intracellular feedbacks (Bergmann et al., 2001, 2002), upregulation of the master transcription factors T-bet and GATA3 (Mariani et al., 2004; Yates et al., 2004) or in the context of cancer and rejection of melanomas (Eftimie et al., 2010). These modeling efforts highlighted the differences between instructive and feedback mechanisms as well as activated pathways in both phenotypes. Other studies solely focused on a single phenotype, such as the work published by Schulz et al. (2009) where the computational model revealed that Th1 differentiation is a two-step process in which the early Th1 cell-polarizing phase is followed by a later phase showing expression of T-bet. Hofer et al. (2002) published a mathematical model showing that GATA-3 transcriptional activation creates a threshold for autoactivation, resulting in two GATA-3 expression states: one for basal expression and one of high expression sustained by its autoactivation.

As new data became available, the increasing complexity of the CD4+ T cell paradigm became evident and new computational approaches were developed to ascertain the regulatory mechanisms controlling differentiation, plasticity, and heterogeneity. van den Ham and de Boer (2008) developed an ODE-based model that describes important regulators and allows for stable switches between several different phenotypes. Other studies focused on the interaction of Th17 and iTreg since Bettelli et al. (2006) described the functional antagonism of Th17 and iTreg. For instance, Hong et al. (2011) constructed a mathematical model of Th17/Treg differentiation that exhibited functionally distinct states, including a RORγt+ FOXP3+. While reductionist approaches have improved our ability to understand small components of the system, studying CD4+ T cell heterogeneity often requires implementing systems approaches and computational methods that can help deciphering complexity. Computational models of CD4+ T cell differentiation and heterogeneity are needed to accurately represent how CD4+ T cells are differentiated and accurately predict sensitivities to determine which pathways and molecules can be most critical to switch from one phenotype to another. A major challenge in systems-level models is the calibration process. Estimation of parameters of large-scale CD4+ T cell differentiation models have proven successful (Carbo et al., 2013b) by following a “divide-and-conquer approach.” This approach is highly useful when parameterizing large models with more than one parameter estimation. First the parameter calibration is divided into smaller parameter estimations: one estimation per phenotype represented in the model. If necessary, other parameter estimations involving specific interactions, such as the Th1/Th2 or the Th17/Treg crosstalk, can be performed. Once parameters are located in a more targeted parameter space, a global parameter estimation is run with all the parameters in the model, allowing us to identify a good global parameter set. These approaches can be easily performed using modeling software such as COPASI (Hoops et al., 2006).

**Figure 1:**
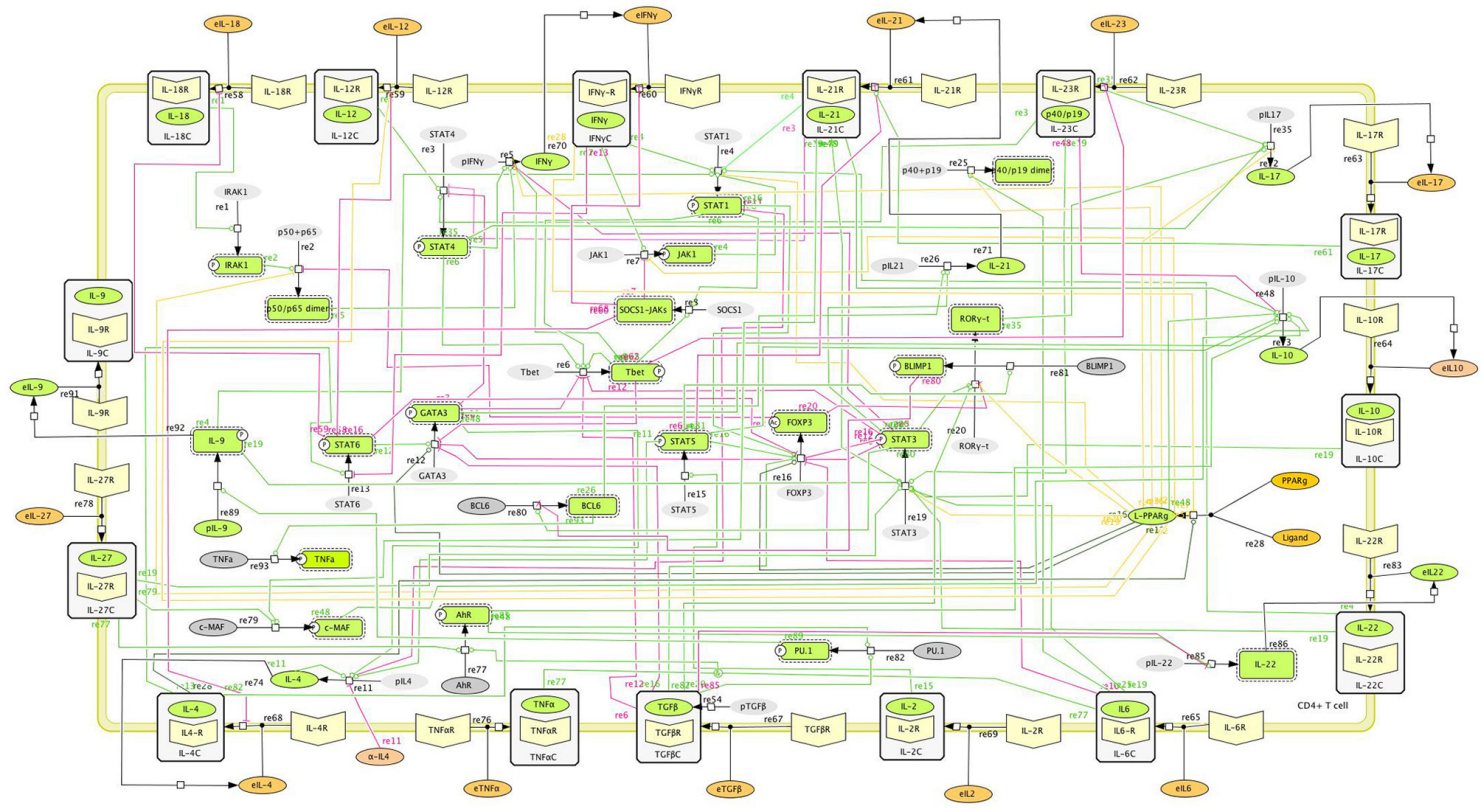
**Main intracellular differentiation pathways of a single CD4+ T cell**. Systems Biology Markup Language (SBML)-compliant network model of CD4+ T cell differentiation, including cytokines, receptors, and intracellular signaling pathways controlling CD4+ T cell fate and function.

The CD4+ T cell differentiation model described in Carbo et al. (2013b) allows the user to have a global understanding with four CD4+ T cell phenotypes represented. The most recent systems biology markup language (SBML)-compliant network (Hucka et al., 2003) provides a structured understanding on different pathways involved in CD4+ T cell differentiation (Figure 1). SBML-based models are indeed highly portable between different simulation platforms. Of note, SBML-based topologies allow standardization in the modeling community and promote cross-transfer of several computational models in an efficient manner. The SBML standards are an essential step toward integrating an ensemble of distributed immunological models (within cells, between cells, at the cell population level, tissue-level, whole organism and human populations).

Another example of CD4+ T cell modeling would be the model by Mendoza and Pardo (2010). In this model, a continuous dynamical system, in the form of a set of coupled ordinary differential equations, was used. Such strategy was then applied to a regulatory network of 36 nodes, representing four CD4+ T cell phenotypes (Th1, Th2, Th17, and Treg). Although this model creates a framework for four phenotypes, the calibration of this larger network, however, was not conducted with experimental data but with default parameters that enabled the differentiation of the four phenotypes, not taking in consideration if reactions occur in a rapid or slow fashion. In addition, the model was not SBML compliant.

Others have explored the contribution of different CD4+ T cell phenotypes to the modulation of immune responses toward *Helicobacter pylori* infection (Carbo et al., 2013a). This study aimed to provide new mechanistic insights on the dynamics of mucosal Th1, Th17, and Treg cells by using both an ODE- and agent-based (ABM) cellular model of the mucosal immune responses during *H. pylori* infection. Alternatively, the logical model strategy has also been used to explore CD4+ T cell differentiation (Mei et al., 2013b; Mendoza, 2013). Mendoza et al. applied either continuous or discrete dynamical systems, regulatory networks of Th1/Th2 or of a combination of different transcription factors adding Th17 and iTreg to represent different states. Even though network modeling has shown to be appropriate, as the production of high-dimensional experimental data is increasingly becoming available, other methods, such as ODE- or agent-based modeling, could help understanding the mechanisms of CD4+ T cell differentiation at the systems level (Hoops et al., 2006; Mei et al., 2012; Wendelsdorf et al., 2012).

## Diving into CD4+ T cell lineages: phenotype or function?

CD4+ T cells form a complex and highly specialized network, representing a major population implicated in mediating host protective and homeostatic responses. However, their excessive or uncontrolled accumulation can also represent a feature in different diseases such as Inflammatory Bowel Disease (IBD) (Abraham and Cho, 2009), Alzheimer's disease (Monsonego et al., 2013), multiple sclerosis (Chitnis, 2007), or allergic disease (Islam and Luster, 2012), among many others. Therefore, their function is closely guided by external signals that are captured from the environment. Also, CD4+ T cells orchestrate immune responses by modulating the function of other cell subsets, such as dendritic cells or macrophages, through secretion of an array of soluble factors, cytokines, and chemokines into the environment. The cytokine profile secreted by each CD4+ T cell will directly depend on which intracellular molecular pathways have been activated, which cytokines are released and how the priming of the single CD4+ T cell has occurred. As an example, IL-6 and TGFβ will activate the Th17 transcriptional machinery, mainly composed by RORγt, RORα, and the phosphorylated form of STAT3. These molecules will activate the transcription of IL-21 and IL-17 and will direct the cell into a Th17 phenotype. However, when a CD4+ T cell is located in an environment rich in TGFβ, lacking IL-6 or other pro-inflammatory cytokines, TGFβ will promote FOXP3 and the phosphorylated STAT5, resulting in the secretion of IL-10 and TGFβ that will activate the regulatory axis. This differentiation dichotomy also depends in part on the T-cell receptor (TCR) engagement and a co-stimulatory signal, frequently involving the CD28 receptor: two basic signals required for a full CD4+ differentiation process. Indeed, Miskov-Kizanov et al. showed how the duration of T cell stimulation through the TCR receptor is a critical determinant of cell date and plasticity by constructing a logic circuit model of TCR signaling pathways in CD4+ T cells (Miskov-Zivanov et al., 2013).

**Figure 2:**
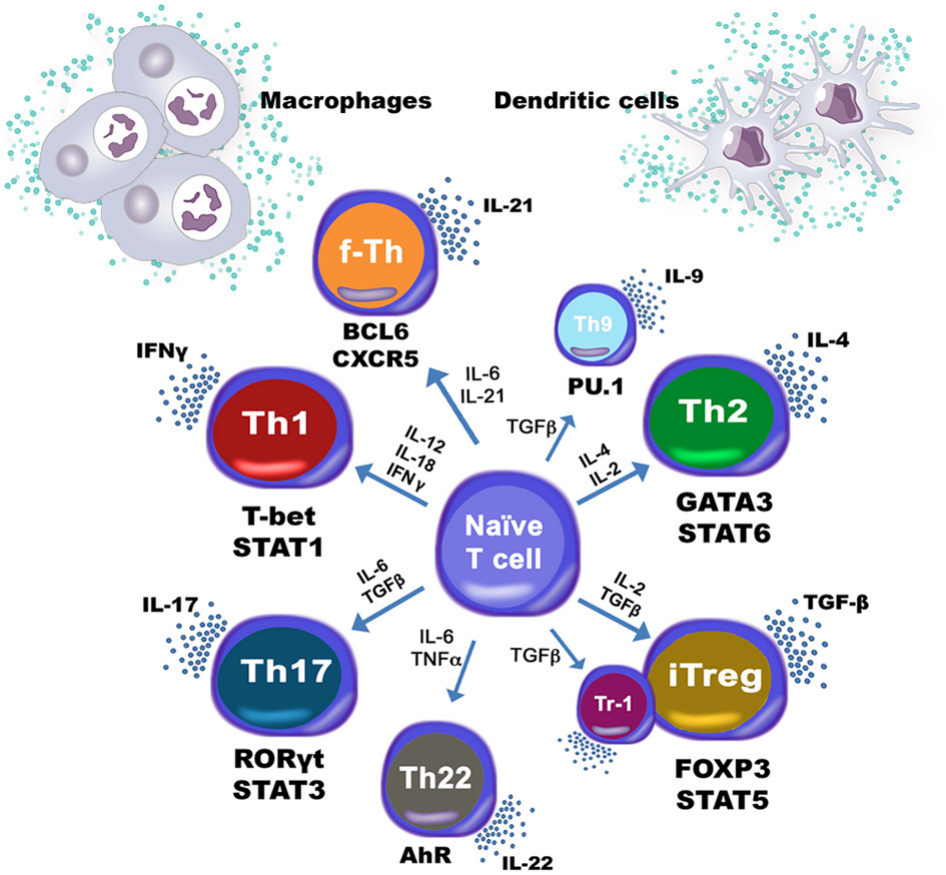
**Heterogeneity of CD4+ T cell subsets**. T helper type 1 (Th1), type 2 (Th2), type 17 (Th17), type 9 (Th9) and type 22 (Th22), Follicular T helper cells (Tfh), and induced regulatory T cells (iTreg) as well as type 1 regulatory T cells (Tr1) are induced based on multiple cytokines being produced by dendritic cells and macrophages among other immune subsets.

CD4+ T cells have a strong predisposition to certain programming and developmental programs enabled by the cytokine environment. However, in the context of disease, where plasticity between phenotypes appears to be the norm, rather than the exception, double positives, such as IFNγ/IL17A often appear in pathological states such as in the context of murine colitis, where the accumulation of IL-17A+ IFNγ+ seems to occur in an IL-23 dependent manner (Ahern et al., 2010). Indeed, IL-23 has been shown to drive CD4+ T helper cell populations into a pathogenic state capable to drive autoimmune population by using passive transfer studies (Langrish et al., 2005), pinpointing IL-23 as a critical player in CD4+ T cell pathogenicity. Moreover, several studies showed that IL-17A could potently induce type 2 diabetes (Arababadi et al., 2010; Jagannathan-Bogdan et al., 2011; Zeng et al., 2012) potentially by modulating the pathogenesis of insulin resistance induced by angiotensin II type 1 receptor (Ohshima et al., 2012) hence increasing the production of renal nitric oxide (Imanishi et al., 2013). Th17 also showed a pleiotropic functionality, since intestine IL-17A+ IL-10+ T cells were found in the small intestine following treatment with anti-CD3 antibody, known to induce an immunosuppressive environment (Esplugues et al., 2011). Furthermore, intestinal epithelial lesions were accentuated in IL-17A null mice (Yang et al., 2008). These implications support a theory, whereby CD4+ T cells are not defined by its inflammatory status but by the functions they accomplish after being exposed to the cytokine milieu. The CD4+ T cell compartment has been demonstrated to be governed, not only by phenotype, but also by function, therefore forcing the distinction between a stable T cell lineage and a T cell differentiation state. Indeed, the ability of a CD4+ T cell to choose a predetermined differentiation program has been shown to be more complex than expected. This determination seems to now bow down to a more functional approach, where CD4+ T cells are not determined by phenotype, but by function, as needed. The functionality of CD4+ T cells as a means of classifying and determining their operational status has already been discussed in O'Connor et al. (2010) and Basu et al. (2013). The traditional view on the CD4+ T cell dogma has now changed into a more comprehensive vision, where the innate immune compartment influences differentiation on CD4+ T cells and not only 2 or 4, but 8 known phenotypes are represented and new phenotypes or states are likely to emerge (Figure 2).

## Deciphering CD4+ T cell plasticity by using computational modeling approaches

Transcription factors, TCR, chemokines, surface receptors, and cytokines determine how CD4+ T cells become activated, maintained and how they can mature into distinguishable featured profiles. However, an increasing understanding on how the mechanisms of differentiation work is revealing increased flexibility and plasticity between different CD4+ T cell phenotypes that allow functional heterogeneity. As discussed above, the functional plasticity between Th1 and Th17 cells resulting in IFNγ+ IL-17A+ CD4+ T cells (Lee et al., 2009; Kurschus et al., 2010) has already been investigated. Indeed, Th17 has been shown to be a very unstable phenotype (Mathur et al., 2006). Functionally, Th17 cells during mucosal inflammation seem significantly different than those Th17 cells involved in regulating homeostasis at the steady state. Whereas IL-17A single positive Th17 cells produce IL-22, which may provide a mechanisms through which Tregs cells reinforce the epithelial barrier (Lin et al., 2014), this same Th17 population can accumulate and produce additional mediators such as IFNγ or GM-CSF during gut inflammatory disorders (Ahern et al., 2010; Codarri et al., 2011; El-Behi et al., 2011). CD4+ T cell plasticity is not only initiated by a change within the intracellular compartment, but also by a change in the extracellular environment. Th1 cells have been demonstrated to acquire plasticity toward a follicular T helper (Tfh)-like phenotype when they encounter a cytokine milieu that is not rich in IL-2 (Liao et al., 2011; Oestreich et al., 2012). Other studies also suggest that early Th1 differentiation is marked by a Tfh cell-like transition highlighting the role of Tbet and STAT4 in mediating these transitions (Nakayamada et al., 2011). The regulatory phenotype iTreg has also been reported to adopt plasticity mechanisms. Several studies have identified, for example, a double RORγt+ FOXP3+ (Lochner et al., 2008; Zhou et al., 2008) that can further differentiate into a pathogenic IL-17-expressing CD4+ T cell (Osorio et al., 2008). These examples illustrate the need for improving our mechanistic understanding at the systems level, where plasticity in the *in vivo* setting needs to be at focus.

Computational methods have also been applied to study the plasticity of CD4+ T cells. Magombedze et al. considered a population plasticity mechanism between Th1 and Th2 during *Mycobacterium avium* infection by using a reduced ODE-based model where the phenotype change of MAP-specific T cells occurred due to differences in the rates of differentiation, proliferation, and death at the site of infection (Magombedze et al., 2014). However, the cellular plasticity involving several intracellular pathways was not represented. In contrast, Pedicini et al. used computational models to analyze the cellular plasticity between Th1 and Th2 cells, extending the regular Tbet/GATA3 plasticity predictions to a broader panel of molecules, involving IRF4, STAT1 and STAT6, MAF, NFAT, and SOCS1 (Pedicini et al., 2010). More comprehensive approaches have also been explored by using extended logical formalisms with Boolean variables to assess the effect of different cytokines in making a CD4+ T cell evolve toward a specific state (Naldi et al., 2010). As a general rule, validation studies are performed to endorse and corroborate the usefulness of computational models. Whereas computational models may be used for *in silico* experimentation, *in vivo* and *in vitro* validation needs to be performed in order to ensure its predictability and prove that the plasticity described *in silico* can be translated into an *in vivo* setting in those cases. To address plasticity *in vivo*, the modeling cycle needs to be completed; first, the model needs to be created based on either available data and/or theory-driven knowledge. Afterwards, calibration procedures need to ensure that a good parameter value set has been found and quality control needs to be run to check that the computational model fully represents our experimental data. Third, *in silico* experimentation, using loss-of-function, overexpression or sensitivity analysis strategies need to be performed. Finally, *in vivo* or *in vitro* validation studies will authenticate the computational model and serve as future calibration data for model refinement. These new approaches are helping immunologists to target novel experiments that will shed some light to the subjective issue of CD4+ T cell plasticity.

The computational CD4+ T cell differentiation landscape has generated several validated studies. We validated experimentally that activation of the transcription factor peroxisome proliferator activated receptor gamma (PPARγ) favored the plasticity of Th17 cells toward iTreg cells, a key prediction of our CD4+ T cell model (Carbo et al., 2013b). This model consisted of 60 differential equations, representing 52 reactions and 93 species, computing the differentiation of a CD4+ T cell into Th1, Th2, Th17, and Treg. The model included cytokines, nuclear receptors and transcription factors that defined fate and function of CD4+ T cells. The first set of computationally derived hypotheses were centered around PPARγ and its modulatory role between Th17 and iTreg. Time course simulations illustrated how PPARγ can trigger plasticity in IL-17A+ producing Th17 cells, causing the system to become a iTreg CD4+ T cell. To validate this prediction, *in vitro* and *in vivo* experiments in the context of an IBD onset were designed with PPARγ null CD4+ T cells as well as with a treatment with pioglitazone, a PPARγ activator. The study presented in Miskov-Zivanov et al. (2013) also validated the interaction of FOXP3 and mTOR following TCR activation by purifying and activating DCs and CD4+ T cells and assessing the expression of different intracellular markers using cell staining and flow cytometry. Another example is the validation of the time-dependent, dual T-bet wave during Th1 differentiation validated using gene expression analysis in CD4+ T cells isolated from wild-type and IFNγ null mice (Schulz et al., 2009).

## Complementarity of theoretical and data-driven models

In computational immunology, often times, the available knowledge about a given set of biological events is used to construct a specific mathematical model. This theoretical approach is therefore directly correlated to the amount of information that is publicly available and the model created upon these pieces of data will only represent the processes delineated within. On the other hand, models can be constructed based solely on analyzing data itself. The increasing availability of high-dimensional data to quantify signaling and cellular responses, together with the novel sequencing technology advancements, is opening a new avenue to use these data-rich datasets to build computational models and help understanding CD4+ T cell differentiation responses. This systems-biology approach, however, can be a double-edged sword: generating high-throughput datasets is part of a big-data strategy, and sometimes, without the appropriate tools, can bring more confusion than understanding to the problem (Bray, 2001). On the other side, this increased availability of data, if used correctly, can streamline the modeling approach, offering a tremendous amount of data for calibration purposes that could allow modelers to build fully calibrated, predictive and extremely comprehensive models that could help generate important hypotheses. These two opposed modeling views can actually be used as a complementary strategy. Theoretical models lack data either for network architecture construction or for model calibration. Data-driven modeling, however, is sometimes confusing, and lack general rules to guide the user and make sense of such big pieces of data. Combining the organization-based approach from theory-driven models with the amount of data and novelty from the data-driven model, highly predictive, hybrid models can be ultimately constructed. In fact, substantial evidence has been shown to understand that the just and only use of data-driven models can represent a trap. The so called “Big Data Hubris” (the often implicit assumption that big data are a substitute, rather than a supplement to, traditional data collection and analysis) already triggered an overestimation of Google's assessment on flu prevalence in 2013 (Lazer et al., 2014). This is a clear example on how data-based and data-driven results were wrongly generated due to the lack of theory underlying unstructured data integration.

The long-standing traditional theory-driven approach has been proven to provide helpful insights on how CD4+ T cells function, where modeling strategies are based on prior biological understanding of the molecular mechanisms involved (Fishman and Perelson, 1999; Hofer et al., 2002; Mendoza, 2006; Klinke, 2007; Hong et al., 2011). However, often times theory-driven modeling is intimately linked to reductionist approaches, since the availability of calibration data can become an issue if building comprehensive networks. Data-driven modeling emerges as a new and complementary approach for multivariate analysis and systems-level analyses. Often times, predictability in computational systems is linked to either the lack of data to construct the computational model or the limitations on the model topology. The combination of data-driven approaches and theoretical strategies may solve these problems therefore promoting the creation of truly predictive models. An example on how to use high-throughput data to construct a CD4+ T cell comprehensive network is the study published by Yosef et al., where they used transcriptional profiling with microarrays at high temporal resolution to build a Th17 induction system (Yosef et al., 2013). In this study, 1291 genes were differentially identified and clustered into 20 groups, depending on their temporal profiles. Another advantage highlighted in this study is the use of modules to explain the processes controlling Th17 differentiation. Four regulatory modules were identified: the positive module that increased IL-17 levels, the negative module that downregulated IL-17, the signature of Th17 genes and signature of other CD4+ T cell subtypes. This work supported the finding of 3 novel key regulators of Th17 function: *Mina*, *Fas*, and *Pou2af1*. Another study where data-driven approaches were taken was the work performed by Ciofani et al., where they combined genome-wide transcription factor occupancy, expression profiling of transcription factor mutants, and transcriptional regulatory network (Ciofani et al., 2012). Integration of several datasets allowed the inference of a Th17 network that highlighted some key regulators to Th17 plasticity, such as *Fosl2*. These two approaches have unveiled novel nodes by using a data-driven approach. However, both networks, which represent static pictures, lack dynamics running on the background. By adding dynamics to the system, a whole new dimension can be added. These data-rich models could be used to determine how the system evolves when a node is knocked-out, or how sensitive are reactions and fluxes to change by a special drug or modulator in a more mechanistic manner. A counterfactual example related to the CD4+ T cell differentiation process is the role of IL-17A in chronic inflammation during IBD. Although it has been reported increased expression of IL-17A during IBD (Fujino et al., 2003) and both IL-17R-deficient mice in TNBS-induced colitis model (Zhang et al., 2006) as well as IL-17A-deficient mice in a DSS-induced colitis model (Ito et al., 2008) were reported to worsen the clinical disease symptoms, some other opposing studies highlighted the protective role of IL-17A production *in vivo* (Ogawa et al., 2004; O'Connor et al., 2009). Very interestingly, a human anti-IL-17A monoclonal antibody to treat Crohn's disease showed that blockade of IL-17A in humans was ineffective and higher rates of adverse events were noted compared with the placebo group (Hueber et al., 2012). In this case, where it is clear there are missing pieces in this puzzle, a combined strategy with both theory-driven and data-driven modeling could shed some light by looking at other players in these intricate and complex interactions.

Data-driven modeling nicely complements and synergizes with theory-driven due to the availability of data for calibration purposes, the potential of discovering novel regulators in the network that have never been described before, and the capability to comprehensively and mechanistically understand complex systems. At the same time, hypotheses extracted from modeling need to be validated to become accepted theories by the community. The combination of theory driven models with data-driven approaches is becoming a strong, useful tool to ensure that the basic knowledge is represented, but at the same time, that novelty and higher predictability is reached. The combination of these two different strategies and multiscalability is now increasing the predictability of very comprehensive models.

## Deterministic vs. stochastic approaches

In complex regulatory schemas, such as the CD4+ T cell differentiation network, gene expression is controlled by transcriptional signals that determine how rapid and how often a specific gene is transcribed. This transcription process, however, depends on other signals and molecules, such as transcription factors and promoter signals that will trigger cell-to-cell variability. Often times, gene transcription is a result of a combination of other signaling cascades, therefore adding not only complexity and variability due to the differential activation of upstream molecules, but also a time delay while the signal molecule concentration either accumulates or decays.

In CD4+ T cell differentiation, variability is a key component of the process. In fact, not all the cells expressing RORγt exhibit IL-17A production even in the presence of the correct inductors TGFβ and IL-6 (Zhou et al., 2008). Furthermore, Guo et al. showed how IL-4 secreting and non-secreting cells from Th2 cultures have a similar probability of producing IL-4 upon subsequent stimulation, implying that there is stochastic element in IL-4 production by stimulated Th2 cells (Guo et al., 2004). Even after assuming that most genes are expressed from both alleles when the transcription machinery is in place, some studies point out that some cytokine genes in T cells are often expressed in a monoallelic manner (Riviere et al., 1998). Alternatively, the transcription rates also vary if agonistic transcription factors are bound (Chen et al., 2011). Given these set of premises, stochastic approaches that add this type of variability within the CD4+ T cell subset can be used to help explain biological variation. In this case, this variability offers a unique way to control regulation, by inducing stimuli but controlling the fraction of cells expressing a specific cytokine.

Deterministic models of CD4+ T cell differentiation are more prevalent than stochastic-based models. Of note, deterministic approaches have unveiled a large amount of findings that relate to single cell behavior. A fraction of these models have focused on the analysis of one phenotype only (Schulz et al., 2009; Gross et al., 2011), and other models have focused on more than one phenotype and the interactions between the resulting states (van den Ham and de Boer, 2008; Gross et al., 2011; Hong et al., 2011; Carbo et al., 2013b). Mariani et al., in contrast, used a stochastic approach to show how an IL-4 stochastic mechanism acting at the chromatin level can be integrated with transcriptional regulation to quantitatively control cell-to-cell variability (Mariani et al., 2010). Furthermore, Santoni et al. used an agent-based model to assess Th1 vs. Th2 fates in the context of hypersensitivity reactions (Santoni et al., 2008). Recently, Mei et al. assessed the role of the IL-6 receptor in controlling the balance between Th17 and iTreg using a novel, web-based stochastic modeling tool (Mei et al., 2013a). Other approaches have used the mathematical formulation of a cell population master equation (CPME) that describes population dynamics and takes into account the major sources of heterogeneity, namely stochasticity in reaction, DNA-duplication, and division, using the Montecarlo algorithm (Stamatakis and Zygourakis, 2010). Manninen et al. (2006) developed several approaches to incorporate stochasticity into deterministic differential equation models, obtaining so-called Itô stochastic differential equations, and applied them to neuronal protein kinase C signal transduction pathway modeling. Even though traditional molecular biology research has tended to composite single cell deterministic models, diversification of T cell fate during CD4+ T cell differentiation implies that the fate of any individual cell may also be acquired stochastically. Therefore, stochastic simulations within the CD4+ T cell differentiation process could help to understand the tight regulation between phenotypes as well as help identify key nodes that, when acting at higher variability, can skew the output of differentiation into a specific differentiation program.

## Application of multiscale modeling to study CD4+ T cell differentiation

**Figure 3:**
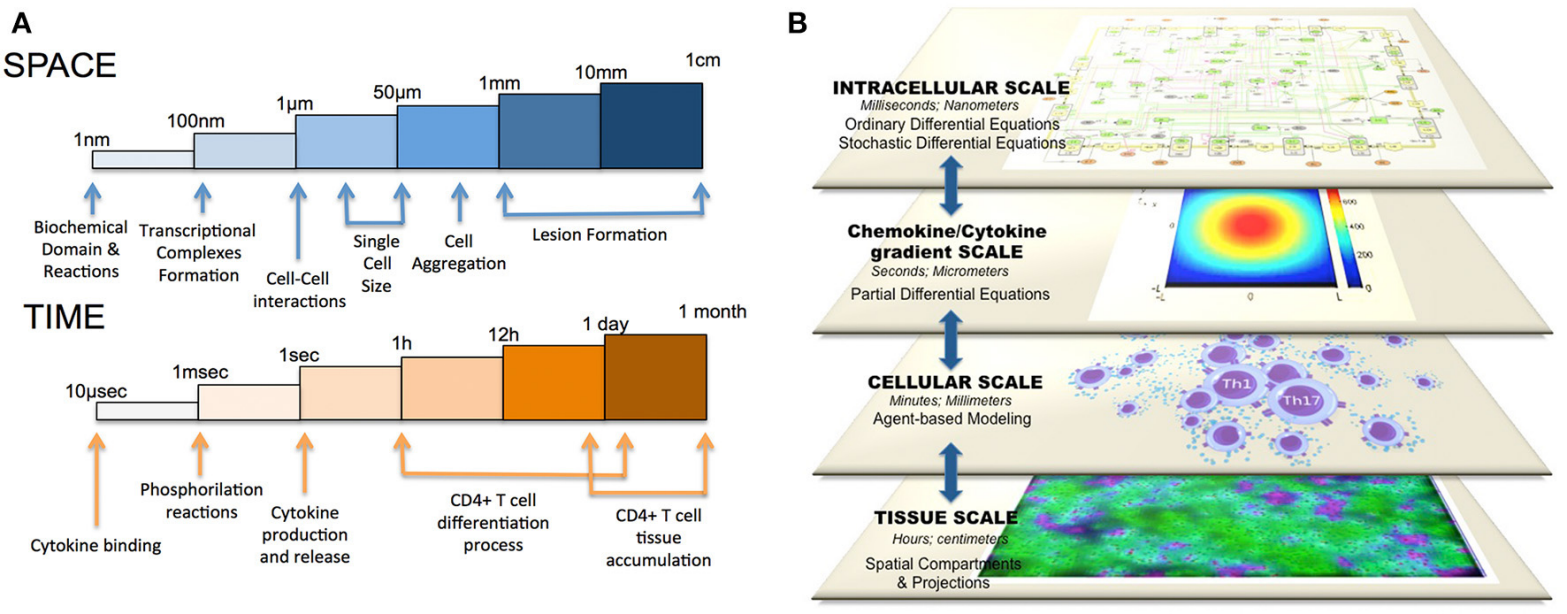
**Multiscale modeling of CD4+ T cell differentiation**. The CD4+ T cell differentiation process comprises **(A)** different spatiotemporal parameters (milliseconds to hours and nanometers to centimeters) as well as **(B)** different scales (intracellular, difussion gradient, cellular, and tissue-level scale).

CD4+ T cell differentiation is a process where a change in the intracellular compartment can tremendously impact the outcome of tissue pathology and clinical disease. Distinct intracellular processes dictate the secretion of chemokines, cytokines, and other soluble factors. These components can, at the same time, modulate other CD4+ T cell nearby by binding to specific receptors. This population effect can modulate other downstream immune subsets that can ultimately affect the formation of lesions at the tissue level. Thus, CD4+ T cell differentiation is not only an intracellular process: population and cellular organization are another major mechanism that may contribute to the change in the dominant phenotype of effector CD4+ T cells during chronic pathologies (Magombedze et al., 2013). Indeed, the mucosal immune system includes hierarchical interactions between cells leading to emerging behaviors with dimensions ranging from nanometers to meters and time scales from nanoseconds to years. The spatiotemporal scales where CD4+ T cells participate can actually range from micro-seconds to months or years and to nanometers to centimeters or meters (Figure 3A). Complex and dynamic information processing networks transfer information across scales in immunity encoding host responses and repair measures. The architecture of such multiscale network also needs to be completely embedded in a comprehensive, integrated system. Because of this flexibility in parameter calibration and sensitivity analyses, Ordinary or Stochastic Differential Equations (ODE or SDE) are ideal candidates to encapsulate and simulate intracellular events. In addition, neural networks have also been used to classify and simulate immune cell subsets (Mei et al., 2013b). In the multiscale setting, these ODE- or SDE-based models would reproduce intracellular CD4+ T cell activation with a release of cytokines and chemokines as a result of the process of differentiation. Partial Differential Equation (PDE) modeling would be a great way to simulate the diffusion reactions of such cytokines in the environment. Ultimately, an agent-based model, adding randomness to the biological system, which helps to better represent responses at the cellular level, would encompass and organize the ODE/SDE models with the PDE simulations by simulating CD4+ T cells as objects that can change its state depending on the cytokine milieu. As a result of these premises, multiscale models are positioned as a comprehensive tool to understand not only the intracellular events happening within the CD4+ T cell compartment at a single cell level, but also understanding the interactions and sensitivities, at the cellular, population and tissue levels, that contribute to disease chronicity, tolerance, or resolution (Figure 3B).

All together, ODE models can calculate the intracellular concentration of different species over time, PDE models could analyze the gradient concentration of cytokines and chemokines secreted by the ODE model, ABM-based models could modulate the cell-cell interactions and spatial compartments could represent the tissue-level scale, including lesion formation. Current experimental techniques are limited in allowing immunologists to quantitatively manipulate immune responses to pathogens in a controlled manner in animal models and to trace events at the tissue level confidently back to specific cellular level interactions and molecular or signaling mechanisms. In a multiscale model, one can test whether mechanisms seen in the experimental context *in vivo* or *in vitro* are plausible explanations for phenomena observed at the clinical level. There have been several previous studies on multiscale modeling in the context of immunity: Sloot and Hoekstra (2010) proposed a multi-scale modeling methodology in computational biomedicine and presented two cases studies. Krinner et al. (2013) coupled an agent-based model of hematopoietic stem cells with an ODE model of granulopoiesis. Also, Klinke (2007) published a multiscale model of dendritic cell education and trafficking in the lung. Some very recent multiscale approaches to study the CD4+ T cell population have been performed in the context of HIV infection (Yeghiazarian et al., 2013) and also in the context of CD4+ T cell migration, signaling, and interaction with the APC compartment (Huang, 2010). Furthermore, Dwivedi et al. recently developed a multiscale systems model of IL-6–mediated immune regulation in Crohn's disease, by integrating intracellular signaling with organ-level dynamics of pharmacological markers underlying the disease (Dwivedi et al., 2014). Santoni et al. (2008) also combined an agent-based model of type I hypersensitivity reactions showing hallmarks of the response to a generic allergen with a gene regulatory network for the switch of Th1/Th2 phenoptypes. Despite all these strategies and studies, there is no comprehensive multiscale model that computes more than two phenotypes of CD4+ T cell differentiation based on the availability of certain factors in the environment and considers more than one scale in the simulation.

Multiscale modeling may also help integrate immune processes and metabolic pathways to build systems-level immunometabolic frameworks. Indeed, T cell metabolism is highly dynamic and has a tremendous impact on the ability of T cells to grow, activate and differentiate (Gerriets and Rathmell, 2012). Glucose metabolism is one of the pathways that has been targeted to explore immunometabolism. One example is the study from Maciver et al. where they found that activation of T cells causes a large increase in glucose transporter 1 (Glut1) expression and surface localization (Maciver et al., 2008). Furthermore, CD28 appeared to promote Akt-independent up-regulation of Glut1 and Akt-dependent Glut1 cell surface trafficking (Jacobs et al., 2008). Multiscale modeling analyses could also help to differentiate which are the metabolic needs to promote specific developmental programs. In fact, effector and regulatory phenotypes have distinct glycolytic and lipid oxidative metabolic programs (Michalek et al., 2011). Pearce et al. reviewed (Pearce, 2010) how activated T cells have an anabolic metabolism, whereas non-proliferating T cells had an opposed catabolic metabolism. Furthermore, autophagy has been found to be essential for T cell survival and proliferation (Pua et al., 2007). Later the same group described how the same process of autophagy may have a physiologically significant role in the clearance of mitochondria in T cells as part of normal T cell homeostasis (Pua et al., 2009), creating a clear link between immunometabolism and T cell function. By using a multiscale strategy, these metabolic programs could be integrated in differentiation simulations and more importantly, the processes could be manipulated to control anti- and pro-inflammatory development in the context of inflammatory diseases. Thus, modeling can be used to quantitatively study dynamic processes located at the interface of immunity and metabolism.

Of note, understanding the mechanisms of CD4+ T cell differentiation and plasticity across scales can lead to the identification of novel therapeutic targets for skewing effector cells into regulatory phenotypes that suppress inflammation. Therefore, multiscale modeling can, indeed, increase predictability and systems-wide mechanistic understanding as to how CD4+ T cells are activated, maintained, and transformed.

## Conclusion

T cell immune responses are extremely heterogeneous and complex. This variability is not fully understood and there are still several questions in regards to CD4+ T cell plasticity and function. Indeed, the issue of what criteria to use to characterize distinct T cell subsets is becoming increasingly complicated. Moreover, the idea that CD4+ T cells are governed by function and not by phenotype is clearly emerging as more double positive and plastic behaviors are being unveiled. The possibility that every helper T cell process is a unique combination of molecules, however, cannot be discarded. This review highlighted how CD4+ T cells have a strong predisposition to certain developmental programs, but it also showed how, at certain times with certain environmental signals, this predisposition is skewed toward another program. Computationally, the plural CD4+ T cell scenario is still a field of interest and active investigation. As new advancements in the understanding of immune responses continue to unfold, computational modeling approaches are likely to be required to comprehensively and systematically investigate mechanisms across spatiotemporal scales and to help integrate diverse data types.

### Conflict of interest statement

The authors declare that the research was conducted in the absence of any commercial or financial relationships that could be construed as a potential conflict of interest.
